# The level of the gender gap in academic publishing varies by country and region of affiliation: A cross-sectional study of articles published in general medical journals

**DOI:** 10.1371/journal.pone.0291837

**Published:** 2023-09-21

**Authors:** Paul Sebo, Joëlle Schwarz

**Affiliations:** 1 University Institute for Primary Care (IuMFE), University of Geneva, Geneva, Switzerland; 2 Center for Primary Care and Public Health, University of Lausanne, Lausanne, Switzerland; University of Montenegro, MONTENEGRO

## Abstract

**Background:**

Women are generally under-represented as authors of publications, and especially as last authors, but this under-representation may not be uniformly distributed across countries. We aimed to document by country and region the proportion of female authors (PFA) in high-impact general medical journals.

**Methods:**

We used PyMed, a Python library that provides access to PubMed, to retrieve all PubMed articles published between January 2012 and December 2021 in the fifty general internal medicine journals with the highest 2020 impact factor according to Journal Citation Reports. We extracted first/last authors’ main country of affiliation for all these articles using regular expressions and manual search, and grouped the countries into eight regions (North/Latin America, Western/Eastern Europe, Asia, Pacific, Middle East, and Africa). We used NamSor to determine first/last authors’ gender and computed the PFA for each country/region.

**Results:**

We retrieved 163,537 publications for first authors and 135,392 for last authors. Gender could be determined for 160,891 and 133,373 publications, respectively. The PFA was 41% for first authors and 33% for last authors, but it varied widely by country (first authors: >50% for eight countries, maximum = 63% in Romania, minimum = 19% in Japan; last authors: >50% for two countries, maximum = 53% in Romania, minimum = 9% in Japan). The PFA also varied by region. It was highest for Eastern Europe (first authors = 53%, last authors = 40%), and lowest for Asia (36% and 29%) and the Middle East (35% and 27%).

**Conclusion:**

We found that the PFA varied widely by country and region, and was lowest in Asia, particularly Japan, and the Middle East. The under-representation of women as authors of publications, especially in these two regions, needs to be addressed and correcting persistent gender discrimination in research should be a top priority.

## Background

Discrimination against female physicians is still a reality in the academic world. The proportion of female physicians has gradually increased over the past few decades [1], to the point where women now account for nearly half of all physicians in OECD (Organization for Economic Cooperation and Development) countries [2]. Yet, they remain a minority in high-level academic positions in medical schools [3, 4], a phenomenon known as the “leaky pipeline” [5, 6]. The reasons for this phenomenon, which is particularly strong in medicine, are diverse and complex. The hypothesis that the gender gap reflects historical differences in unequal access to education did not prove sufficient, as the gap did not naturally evaporate with the increase in the number of women entering the profession since the 1970s [7].

The “leaky pipeline” is rather explained by two interconnected aspects. The first is the time investment required for academic medicine and geographical mobility, which together lead to poor work-life balance and career uncertainty [8–10]. The second is a climate of sexism, sexual harassment and gender discrimination that is particularly strong in clinical settings, because they are highly hierarchical (across positions and professions), male-dominated and include dependent relationships (teacher-learners) [11–13]. Women do not pursue academic careers because of the discrimination they face in terms of maternity leave and family commitments, differential access to mentoring, low levels of acceptance of flexible working and perceived explicit gender bias [14].

As part of and a consequence of the “leaky pipeline” phenomenon, women are generally under-represented as authors of publications, and particularly as last authors (the last author position is traditionally reserved for the principal investigator in health science research). While the proportion of female authors (PFA) in general medical journals is about 40% for first authorship [15, 16], it is only about one-third for last authorship [16, 17].

This under-representation of women authors does not seem to be uniformly distributed across countries. In a recent study that included 767 articles, we found that the PFA was particularly low for publications from Asia, South America and Africa (27% for Asia and 29% for South America and Africa) [18]. In addition to a small sample size and the fact that the analysis focused on a single year (i.e., 2016), this study examined only first authors’ gender. It is likely that the picture is even worse for last authorship. Identifying possible variations by country and/or region of affiliation in the level of the gender gap in academic publishing would raise awareness of this issue among national health research institutions so that they may implement measures to promote equal opportunities in access to academic positions.

Implementing effective measures to increase the representation of women as authors of publications is crucial, not only to improve the academic prospects of female researchers, but also to enable better patient care [19]. Sex and gender are major determinants of health, yet they are often overlooked in research [20, 21]. Failure to conduct sex- and gender-specific analyses can thus lead to harmful consequences for patients, for example in the field of pharmacology [19]. It is now well known that the pharmacokinetics and pharmacodynamics of drugs differ by sex, which can result in different adverse event profiles and greatly influence treatment outcomes [22, 23]. Interestingly, it was shown that female researchers were more likely to include such analyses in their studies [24–26]. With increased representation of women in research, it is therefore expected that the number of studies adhering to the recommendations for reporting and analyzing results by sex and gender will also increase.

To identify possible geographic variations in the level of the gender gap in academic publishing, we conducted a study to document by country and region the PFA for first and last authorship in fifty high-impact general medical journals. We hypothesize that these proportions vary widely across countries depending on the socio-cultural context. A secondary objective was to assess whether there was an association between the Gender Inequality Index (GII) for the countries selected for the study and the proportion of female authors in those same countries.

## Methods

### Selection of journals and publications, and data extraction

We retrieved all PubMed articles published between January 2012 and December 2021 (i.e., over a 10-year period) in the fifty general internal medicine journals with the highest 2020 impact factor according to *Journal Citation Reports* (category = "general internal medicine"; journal #1 = "New England Journal of Medicine", journal #50 = "Military Medical Research"). The list of journals with their 2020 *Journal Citation Reports* impact factor is shown in Table 1. The extraction was done with PyMed, a Python library that provides access to PubMed with the standard PubMed query language (https://github.com/gijswobben/pymed/blob/9c26bff758ced28ce9b794a300d1a8bc788543db/README.md). The database used for the study contained the following information for each article: PubMed identification number (PMID), journal, date of publication, and first name, last name and affiliation for each author.

**Table 1:** List of the fifty general internal medicine journals included in the study, with their 2020 impact factor (according to *Journal Citation Reports*).

N	Journal name	2020 impact factor
1	New England Journal of Medicine	91.253
2	Lancet	79.323
3	JAMA	56.274
4	Nature Reviews Disease Primers	52.329
5	British Medical Journal	39.890
6	Annals of Internal Medicine	25.391
7	Lancet Digital Health	24.519
8	JAMA Internal Medicine	21.873
9	Journal of Cachexia Sarcopenia and Muscle	12.910
10	PLOS Medicine	11.069
11	Cochrane Database of Systematic Reviews	9.289
12	Journal of Internal Medicine	8.989
13	BMC Medicine	8.775
14	Journal of Travel Medicine	8.490
15	JAMA Network Open	8.485
16	Canadian Medical Association Journal	8.262
17	Medical Journal of Australia	7.738
18	Mayo Clinic Proceedings	7.619
19	Amyloid–Journal of Protein Folding Disorders	7.141
20	Translational Research	7.012
21	Deutsches Arzteblatt International	5.594
22	Medical Clinics of North America	5.456
23	British Journal of General Practice	5.386
24	Journal of the Royal Society of Medicine	5.344
25	Panminerva Medica	5.197
26	Annals of Family Medicine	5.166
27	Journal of General Internal Medicine	5.128
28	Frontiers in Medicine	5.093
29	American Journal of Preventive Medicine	5.043
30	American Journal of Medicine	4.965
31	Journal of Personalized Medicine	4.945
32	Minerva Medica	4.806
33	Palliative Medicine	4.762
34	Annals of Medicine	4.709
35	European Journal of Clinical Investigation	4.686
36	American Journal of Chinese Medicine	4.667
37	European Journal of Internal Medicine	4.624
38	British Medical Bulletin	4.291
39	Journal of Clinical Medicine	4.242
40	Preventive Medicine	4.018
41	Postgraduate Medicine	3.840
42	DM Disease-a-Month	3.800
43	Pain Medicine	3.750
44	International Journal of Medical Sciences	3.738
45	Diagnostics	3.706
46	Journal of Urban Health	3.671
47	Journal of Pain and Symptom Management	3.612
48	Journal of Translational Internal Medicine	3.451
49	Internal and Emergency Medicine	3.397
50	Military Medical Research	3.329

After transferring the database into STATA, we extracted the main country of affiliation of first and last authors using regular expressions. Regular expressions, also known as regex, are sequences of characters that define a search pattern. They are used in various programming languages and text editors to perform advanced string matching and manipulation tasks. We added a manual search for publications for which the author’s country of affiliation was missing, based on information provided by PubMed (e.g., university name). We then grouped the countries into eight regions (North and Latin America, Western and Eastern Europe, Asia, Pacific, Middle East, and Africa).

We also extracted for each country the 2021 Gender Inequality Index (GII) [27]. This index is a composite measure that reflects per country the gender-related disadvantages in three dimensions, namely reproductive health (i.e., maternal mortality ratio and adolescent birth rate), empowerment (i.e., access to secondary education, access to parliamentary seats) and the labor market (i.e., participation rate). It shows the loss of potential human development due to the inequality between women’s and men’s achievements in these dimensions. It ranges from 0, where women and men are doing equally well, to 1, where one gender is doing the worst in all dimensions measured. There are five steps to calculating this index, which are described in a technical note (http://hdr.undp.org/sites/default/files/hdr2022_technical_notes.pdf).

### Determination of authors’ gender

We used NamSor (https://namsor.app) to infer first and last authors’ gender. NamSor is a gender detection tool that was recently shown to be one of the two most accurate tools, along with Gender API (https://gender-api.com), for determining the gender of individuals from their first and last names [28]. Its use is simple. After uploading a database in Excel, CSV or text format (https://namsor.app/csv-excel-tool), the file is completed with a "gender" column and three additional columns related to the precision of the estimate: "score", "probabilityCalibrated" and "genderScale". The parameter labeled "probabilityCalibrated" is the most useful for assessing the precision of the estimate, with a probability of one meaning that the precision is 100%. Additional information about NamSor and these parameters is available elsewhere [28].

### Ethical approval

Since this study did not involve the collection of individual health-related data, it did not require ethical review, according to current Swiss law.

### Statistical analyses

We computed for each country and region the PFA for first and last authorship, overall and for 2012–2016 and 2017–2021. We also calculated the Spearman correlation between the PFA and the GII for first and last authorship. For this analysis, we included all countries with more than 50 publications during the period under review (67 countries for first authors and 61 countries for last authors).

We used logistic regressions adjusted for impact factor, year of publication and intra-cluster correlations within journals to determine whether the differences in proportions were statistically significant. We repeated the analyses with two subsamples created by including only publications for which gender was determined with >60% and >80% accuracy (i.e., “probabilityCalibrated” >60% and >80%, respectively), as recommended in other studies [15–17]. The statistical significance was set at a two-sided p-value of ≤0.05. All analyses were carried out with STATA 15.1.

## Results

We retrieved 202,092 PubMed publications. After removing publications by editors or organizations and those without author affiliation information, we obtained 163,537 publications for first authors and 135,392 for last authors. Gender could be determined for >98% of publications (160,891 and 133,373, respectively). As shown in Table 2, the most productive countries were the USA (32% of publications) and the UK (17%). Together, these two countries accounted for almost half of the publications in our study. With 6901 publications, China was only in sixth place.

**Table 2:** Proportion of publications first/last authored by women in fifty high impact general internal medicine journals (2012–2021), and 2021 Gender Inequality Index (GII). The data are presented for countries with more than 50 publications.

Country	2021 Gender Inequality Index	Number of publications by main country of affiliation of first authors (%)	Number of publications by main country of affiliation of last authors (%)	Number of publications first authored by women (%)^a^	Number of publications last authored by women (%)^a^
Overall	NA	160891 (100)	133373 (100)	65302 (40.6)	44072 (33.0)
USA	0.179	50635 (31.5)	44151 (33.1)	20237 (40.0)	14701 (33.3)
UK	0.098	26817 (16.7)	22225 (16.7)	11799 (44.0)	8652 (38.9)
Canada	0.069	10434 (6.5)	8355 (6.3)	4228 (40.5)	2808 (33.6)
Australia	0.073	8354 (5.2)	6547 (4.9)	3939 (47.2)	2484 (37.9)
Italy	0.056	7323 (4.6)	6093 (4.6)	2642 (36.1)	1789 (29.4)
China	0.192	6901 (4.3)	5641 (4.2)	3019 (43.8)	2202 (39.0)
Germany	0.073	4956 (3.1)	3551 (2.7)	1555 (31.4)	729 (20.5)
Spain	0.057	3700 (2.3)	3125 (2.3)	1617 (43.7)	935 (29.9)
France	0.083	3385 (2.1)	2802 (2.1)	1217 (36.0)	746 (26.6)
Netherlands	0.025	3105 (1.9)	2305 (1.7)	1596 (51.4)	765 (33.2)
Japan	0.083	2950 (1.8)	2643 (2.0)	567 (19.2)	235 (8.9)
Korea	0.067	2772 (1.7)	2504 (1.9)	840 (30.3)	489 (19.5)
Taiwan	NA	2386 (1.5)	2124 (1.6)	827 (34.7)	600 (28.3)
Switzerland	0.018	2151 (1.3)	1716 (1.3)	849 (39.5)	469 (27.3)
India	0.490	1923 (1.2)	1557 (1.2)	719 (37.4)	523 (33.6)
Israel	0.083	1801 (1.1)	1510 (1.1)	624 (34.7)	435 (28.8)
Poland	0.109	1799 (1.1)	1671 (1.3)	999 (55.5)	667 (39.9)
Denmark	0.013	1348 (0.8)	1112 (0.8)	578 (42.9)	296 (26.6)
Sweden	0.023	1229 (0.8)	1047 (0.8)	488 (39.7)	346 (33.1)
Belgium	0.048	1178 (0.7)	955 (0.7)	476 (40.4)	283 (29.6)
Brazil	0.390	1001 (0.6)	665 (0.5)	448 (44.8)	245 (36.8)
Austria	0.053	864 (0.5)	701 (0.5)	263 (30.4)	153 (21.8)
New Zealand	0.088	840 (0.5)	554 (0.4)	372 (44.3)	201 (36.3)
Greece	0.119	818 (0.5)	693 (0.5)	279 (34.1)	203 (29.3)
Ireland	0.074	815 (0.5)	603 (0.5)	370 (45.4)	194 (32.2)
Norway	0.016	620 (0.4)	496 (0.4)	273 (44.0)	175 (35.3)
South Africa	0.405	618 (0.4)	407 (0.3)	297 (48.1)	181 (44.5)
Singapore	0.040	593 (0.4)	491 (0.4)	248 (41.8)	161 (32.8)
Finland	0.033	563 (0.4)	391 (0.3)	261 (46.4)	147 (37.6)
Portugal	0.067	560 (0.4)	480 (0.4)	324 (57.9)	211 (44.0)
Hong Kong	NA	547 (0.3)	425 (0.3)	200 (36.6)	127 (29.9)
Romania	0.282	527 (0.3)	491 (0.4)	332 (63.0)	261 (53.2)
Turkey	0.272	475 (0.3)	383 (0.3)	184 (38.7)	97 (25.3)
Iran	0.459	410 (0.3)	270 (0.2)	149 (36.3)	55 (20.4)
Malaysia	0.228	410 (0.3)	319 (0.2)	176 (42.9)	102 (41.0)
Mexico	0.309	410 (0.3)	249 (0.2)	190 (46.3)	110 (34.5)
Lebanon	0.432	384 (0.2)	316 (0.2)	157 (40.9)	111 (35.1)
Thailand	0.333	366 (0.2)	238 (0.2)	159 (43.4)	82 (34.5)
Russia	0.203	257 (0.2)	250 (0.2)	133 (51.8)	89 (35.6)
Saudi Arabia	0.247	247 (0.2)	169 (0.1)	53 (21.5)	24 (14.2)
Peru	0.380	199 (0.1)	143 (0.1)	75 (37.7)	38 (26.6)
Czech Republic	0.120	197 (0.1)	155 (0.1)	64 (32.5)	30 (19.4)
Pakistan	0.534	174 (0.1)	107 (0.1)	56 (32.2)	31 (29.0)
Chile	0.187	168 (0.1)	131 (0.1)	55 (32.7)	36 (27.5)
Hungary	0.221	162 (0.1)	115 (0.1)	53 (32.7)	30 (26.1)
Colombia	0.424	160 (0.1)	93 (0.1)	49 (30.6)	32 (34.4)
Egypt	0.443	159 (0.1)	111 (0.1)	46 (28.9)	44 (39.6)
Argentina	0.287	135 (0.1)	88 (0.1)	46 (34.1)	28 (31.8)
Niger	0.611	135 (0.1)	58 (0.04)	45 (33.3)	11 (19.0)
Croatia	0.093	124 (0.1)	91 (0.1)	58 (46.8)	36 (39.6)
Kenya	0.506	116 (0.1)	101 (0.1)	48 (41.4)	36 (35.6)
Serbia	0.131	114 (0.1)	82 (0.1)	64 (56.1)	39 (47.6)
Uganda	0.530	111 (0.1)	72 (0.1)	41 (36.9)	22 (30.6)
Mali	0.613	109 (0.1)	70 (0.1)	52 (47.7)	16 (22.9)
Bangladesh	0.530	107 (0.1)	83 (0.1)	35 (32.7)	28 (33.7)
Jordan	0.471	96 (0.1)	70 (0.1)	38 (39.6)	19 (27.1)
Vietnam	0.296	94 (0.1)	62 (0.1)	29 (30.9)	18 (29.0)
Palestine (State of)	NA	87 (0.1)	80 (0.1)	40 (46.0)	31 (38.8)
Slovenia	0.071	82 (0.1)	75 (0.1)	42 (51.2)	31 (41.3)
Qatar	0.220	74 (0.1)	75 (0.1)	20 (27.0)	11 (14.7)
Indonesia	0.444	62 (0.04)	NA	30 (48.4)	NA
Lithuania	0.105	62 (0.04)	55 (0.04)	29 (46.8)	28 (50.9)
Bahrain	0.181	58 (0.04)	NA	16 (27.6)	NA
Malawi	0.554	58 (0.04)	NA	26 (44.8)	NA
Nepal	0.452	57 (0.04)	NA	21 (36.8)	NA
Tanzania	0.560	53 (0.03)	NA	22 (41.5)	NA
Bulgaria	0.210	50 (0.03)	NA	26 (52.0)	NA

^a^ both p-values <0.001 (logistic regressions adjusted for impact factor, year of publication and intra-cluster correlations within journals)

**Fig 1:**
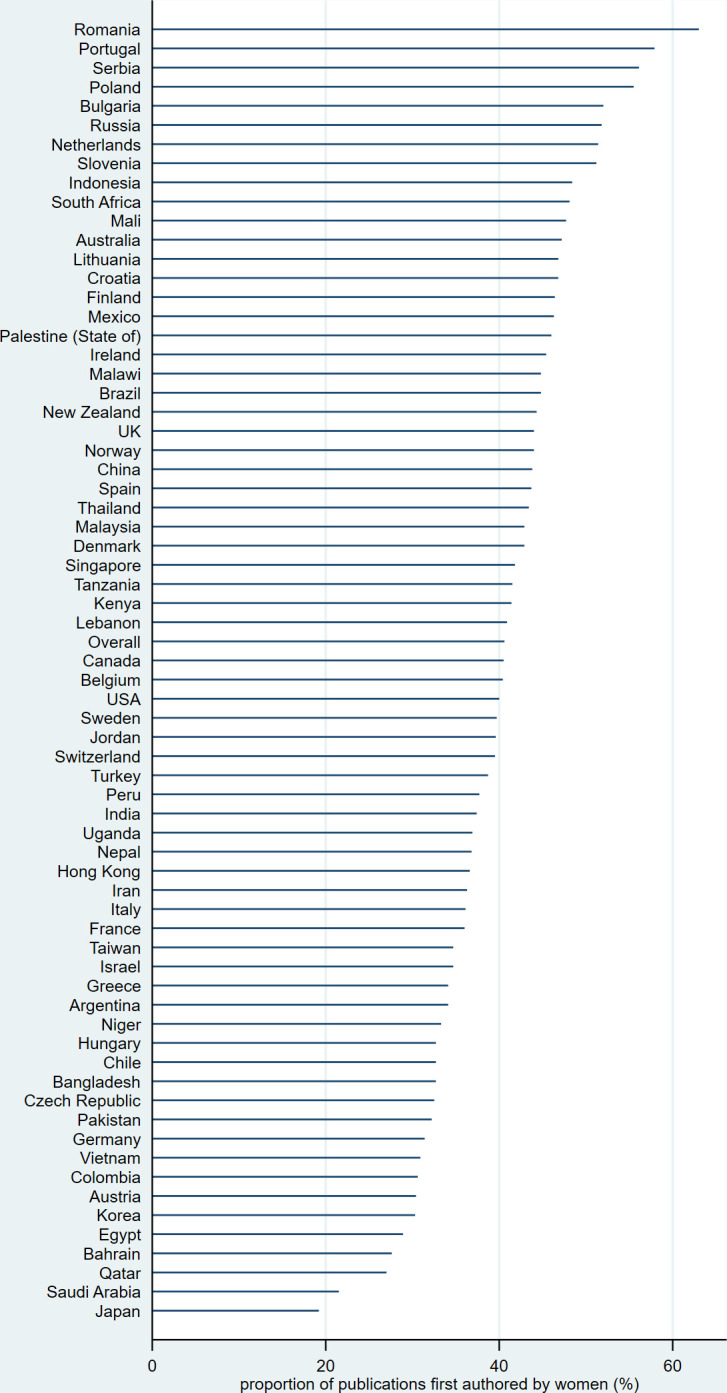
Proportion of publications first authored by women, by main country of affiliation. Countries are sorted by descending proportion.

**Fig 2:**
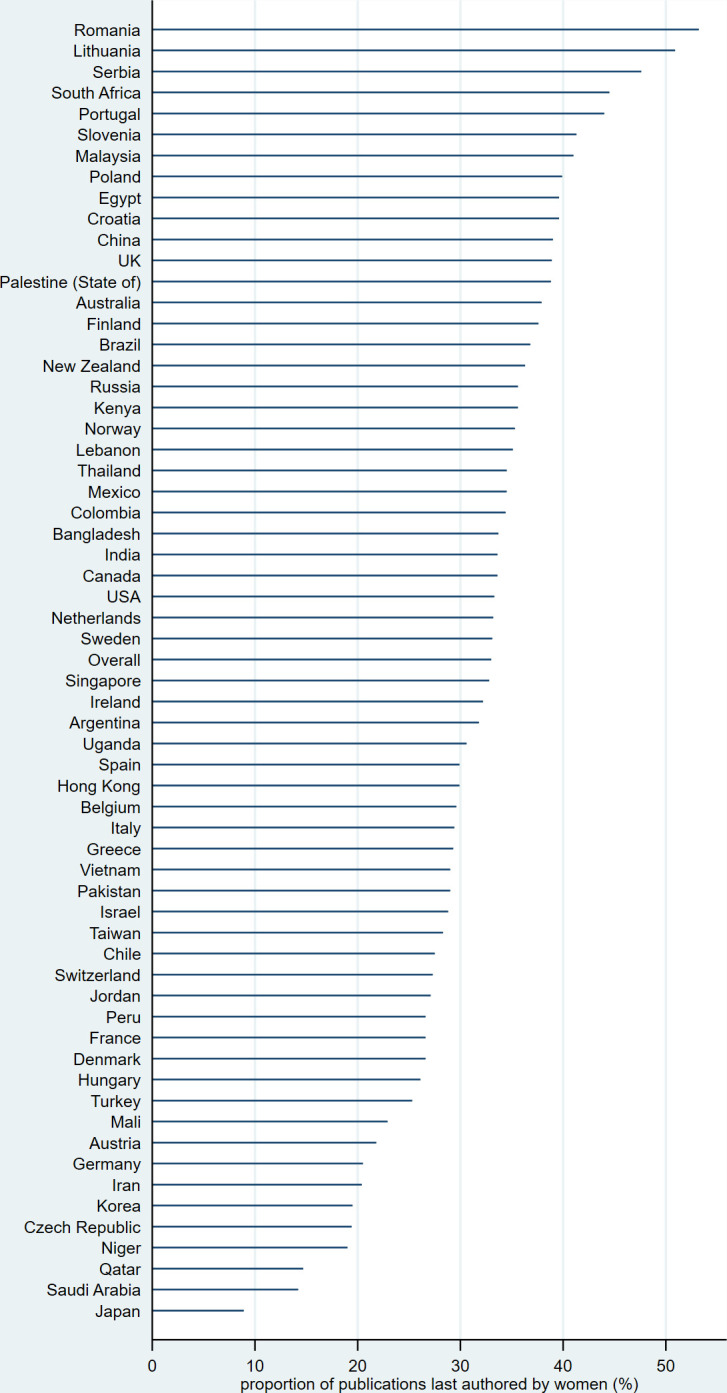
Proportion of publications last authored by women, by main country of affiliation. Countries are sorted by descending proportion.

The PFA was 41% for first authors and 33% for last authors, but it varied widely by country. Table 2, and Figs 1 and 2 show the proportion of publications first/last authored by women for countries with at least 50 publications during the period under review. The proportion for first authors exceeded 50% for eight countries and was highest in Romania (63%), but was only 19% for Japan (adjusted p-value <0.001). The proportion for last authors was over 50% for two countries (Romania and Lithuania) and only 9% for Japan (adjusted p-value <0.001). As Table 2 shows, the most gender-equal countries according to the GII are the Northern European countries and Switzerland (#1: Denmark, #2: Norway, #3: Switzerland, #4: Sweden, #5: The Netherlands). The correlation between the PFA and the GII was weak for the first authors (Spearman’s rho = -0.13, p-value = 0.31) and negligible for the last authors (Spearman’s rho = -0.01, p-value = 0.92).

**Fig 3:**
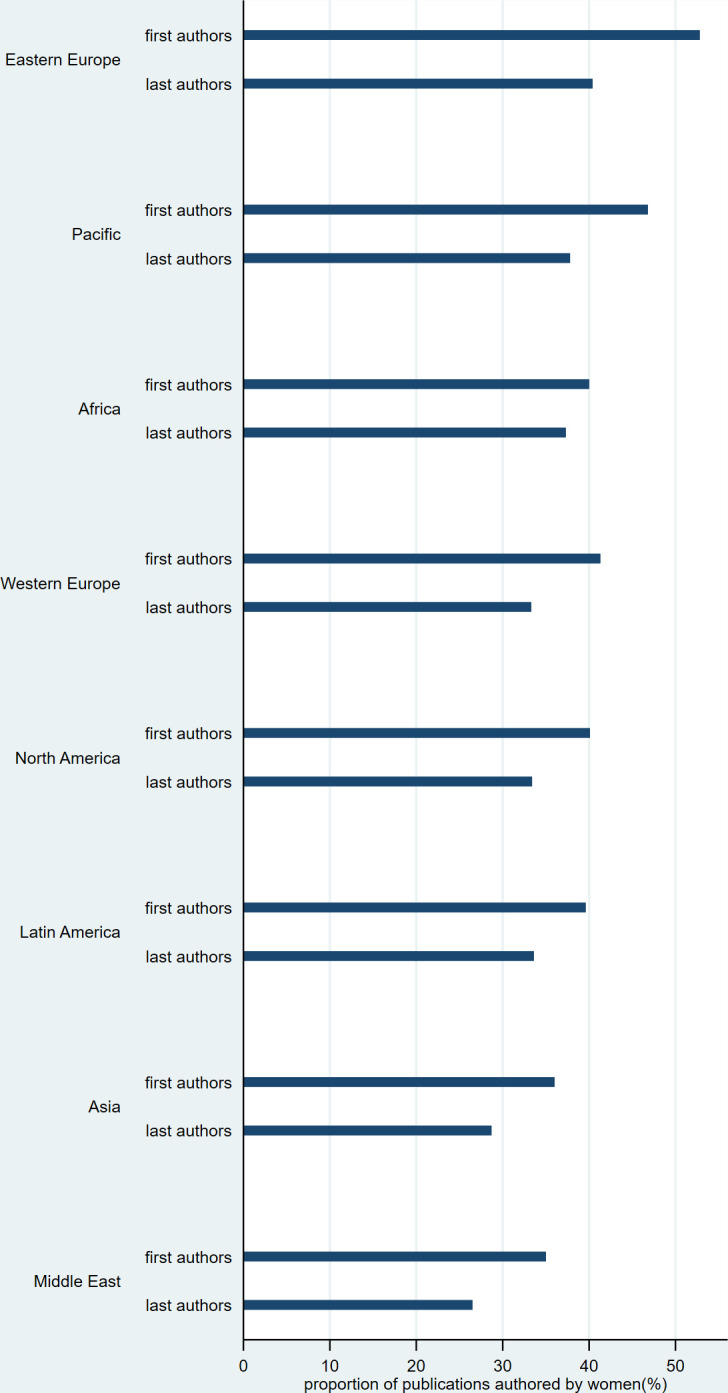
Proportion of publications first/last authored by women, by main region of affiliation. Regions are sorted by descending proportion (= mean proportion between first and last authors).

The PFA also varied by region (Table 3 and Fig 3). The differences were statistically significant for both first and last authors (adjusted p-values <0.001). The proportion was highest for Eastern Europe (first authors = 53%, last authors = 40%), and lowest for Asia (36% and 29%) and the Middle East (35% and 27%). Overall, the PFA was slightly higher for 2017–2021 (42% and 34%) than for 2012–2016 (39% and 31%). It increased for six regions for first authors and five regions for last authors. The results were similar with the subsamples (accuracy>0.6 and accuracy>0.8).

**Table 3:** Proportion of publications first/last authored by women in fifty high impact general internal medicine journals (2012–2021). The data are presented by main region of affiliation, both overall and by time period (2012–2016 & 2017–2021).

Region	Number of publications (%)	Number of publications authored by women (%)^a^	Number of publications authored by women for gender inference accuracy>0.6 (%)^a^	Number of publications authored by women for gender inference accuracy>0.8 (%)^a^	Number of publications authored by women in 2012/2016 (%)^a^	Number of publications authored by women in 2017/2021 (%)^a^
First authors						
Overall	160891 (100)	65302 (40.6)	61732 (40.6)	57103 (40.9)	22470 (38.5)	42832 (41.8)
North America	61069 (38.0)	24465 (40.1)	23541 (39.9)	22161 (39.9)	8534 (36.5)	15931 (42.3)
Western Europe	59588 (37.0)	24636 (41.3)	24101 (41.1)	23448 (41.7)	9003 (40.1)	15633 (42.1)
Asia	19520 (12.1)	7029 (36.0)	5221 (35.4)	3103 (33.8)	1753 (36.6)	5276 (35.8)
Pacific	9227 (5.7)	4317 (46.8)	4162 (46.8)	3915 (47.0)	1999 (44.4)	2318 (49.1)
Middle East	3763 (2.3)	1318 (35.0)	1249 (35.3)	1136 (35.1)	412 (31.7)	906 (36.8)
Eastern Europe	3556 (2.2)	1879 (52.8)	1867 (53.0)	1845 (53.2)	142 (41.0)	1737 (54.1)
Latin America	2329 (1.5)	923 (39.6)	901 (39.3)	880 (39.6)	330 (39.8)	593 (39.6)
Africa	1839 (1.2)	735 (40.0)	690 (40.5)	615 (39.9)	297 (38.1)	438 (41.3)
Last authors						
Overall	133373 (100)	44072 (33.0)	41437 (32.7)	38763 (32.9)	11420 (31.4)	32652 (33.7)
North America	52506 (39.4)	17509 (33.4)	16910 (33.1)	16109 (33.2)	4616 (29.5)	12893 (35.0)
Western Europe	48435 (36.3)	16124 (33.3)	15703 (33.1)	15173 (33.0)	4638 (33.9)	11486 (33.0)
Asia	16340 (12.2)	4688 (28.7)	3220 (26.0)	2134 (25.2)	733 (29.0)	3955 (28.6)
Pacific	7125 (5.3)	2691 (37.8)	2629 (37.7)	2514 (37.4)	978 (34.6)	1713 (39.9)
Middle East	3008 (2.3)	798 (26.5)	759 (26.1)	698 (25.7)	167 (21.6)	631 (28.2)
Eastern Europe	3148 (2.4)	1273 (40.4)	1263 (40.5)	1248 (40.8)	49 (29.3)	1224 (41.1)
Latin America	1601 (1.2)	538 (33.6)	530 (33.5)	514 (33.4)	112 (33.7)	426 (33.6)
Africa	1210 (0.9)	451 (37.3)	423 (36.5)	373 (36.2)	127 (35.1)	324 (38.2)

^a^ all p-values <0.001 (logistic regressions adjusted for impact factor, year of publication and intra-cluster correlations within journals)

## Discussion

### Main findings

In this study, we examined the gender of first and last authors by country and region of affiliation for all PubMed articles published between January 2012 and December 2021 in the fifty general internal medicine journals with the highest impact factor. We found that the most productive countries in terms of number of publications were the USA and the UK. We also found that, overall, the proportion of female authors (PFA) was 41% for first authors and 33% for last authors. However, the PFA varied widely by country and region, and was lowest in Asia, particularly Japan, and the Middle East. For these two regions, the PFA for first and last authorship was about 5% less in absolute value than the average proportion. Finally, we found that the Gender Inequality Index (GII) provided by the United Nations was only weakly correlated with the PFA.

### Comparison with existing literature

The PFA calculated from our data (i.e., 41% for first authors and 33% for last authors) are similar to those from three other studies: 41–43% for first authorship and 32–34% for last authorship [15–17].

The fact that the representation of women as authors of publications varies according to the countries or regions examined was already highlighted in a recent study by our research group [18]. However, the study had a small sample size, and the conclusions that could be drawn from the data were therefore limited. In this previous study, the PFA was lowest for publications from Asia (27%). These regional differences are not surprising if they are linked to the two aspects that lead to the ’leaky pipeline’ phenomenon, namely the academic research performance expectations and the sexist and gender discrimination climate. Academic research performance expectations (i.e., conducting competitive research, teaching and clinical activities in a short and uninterrupted period of time, including mobility for networking) are highly dependent on the organization of the work-life balance. The availability of childcare and elderly care facilities varies considerably from country to country. They are prerequisites for equal opportunities for men and women to pursue an academic career while leading a family life [29]. In the absence of such facilities, given that caring roles are traditionally assigned to women, it will be difficult for women to maintain a dual career.

Similarly, countries and regions experienced different women’s empowerment movements, with different political and institutional impacts, such as laws prohibiting gender discrimination in the workplace and better representation of women in politics resulting in support for the development and funding of equality measures. Socialist political systems were generally proactive in providing “social infrastructure” and equal access to education and labor, and this may be reflected in the higher PFA in Eastern Europe for example, or in China compared to Japan for the Asian region. The case of India illustrates how women’s access to education does not necessarily translate into equal access to labor and leadership positions. In India, the representation of women with PhDs in STEM (i.e., science, technology, engineering and mathematics) is high, but their academic careers in this field are severely hampered by gender discrimination in the workplace, which intersects with other existing forms of social hierarchy such as caste/class. As Gupta described, the judgement of academic excellence and subsequent merit (such as the possibility of geographical mobility, building networks with other scientists) is gendered and strongly disadvantages women scientists who "lack such contacts due to Indian segregation norms, gender stereotyping, lack of mobility and dual burden [after marriage]" [30]. The particularly low PFA in Japan may be explained by institutional discrimination against women in medical school entrance examinations [31].

The increase in PFA between 2012–2016 and 2017–2021 observed in almost all countries may be linked to the third feminist wave that, in many regions, denounced discrimination against women in the workplace, including in clinical settings. The weak correlation found between the GII and the PFA may also be explained by the “leaky pipeline” effect. While the overall gender gap has decreased in most countries over the past decades, the factors described above that hinder women’s access to academic careers are either not yet addressed or have not yet produced tangible effects.

### Implications for practice and research

Numerous studies demonstrated that gender discrimination persists within academic publishing and other areas of academia. For example, in a previous study we found that papers authored by women were less likely to be published in high-impact medical journals compared to papers authored by men, even when controlling for various factors [15]. In a second study we also found that papers authored by women were cited less often than papers authored by men [32]. Similarly, Moss-Racusin et al. conducted an experiment revealing that both male and female faculty members evaluated identical job applications more favorably when the applicant’s gender was male [33]. Finally, Knobloch-Westerwick et al. examined the influence of authors’ gender on the perceived quality of scientific publications [34]. They found that publications from male authors were associated with greater scientific quality. These findings strongly support the notion that gender discrimination persists within academia and affects women’s opportunities for recognition and advancement.

However, recent evidence on the “leaky pipeline” phenomenon, including research on sexism and sexual harassment in institutions [11], and the reforms and initiatives that are beginning to be taken by medical schools and research institutes in various countries to address the issue may give hope for change in the future. Certainly, a number of measures can be effective in reducing the gender gap in publication and, more generally, the inequalities faced by women in academic medicine (Box 1). By implementing these measures, the academic community can work towards mitigating gender disparities in publishing and creating a more inclusive and equitable environment for researchers. Universities and academic organizations should be at the forefront of promoting and supporting female researchers throughout their careers, for example by providing sufficient time for research or by increasing the visibility of their studies, whether in terms of media coverage or conference participation. Furthermore, the way researchers are evaluated and compared to each other should be questioned and other indicators developed. Indeed, the indicators commonly used to evaluate researchers, such as the number of publications, the number of citations and the h-index, are very reductive and do not always reflect the involvement of researchers or the quality of their research. Initiatives such as the San Francisco Declaration on Research Assessment (DORA) for a fairer evaluation of scientific research outputs are therefore promising (https://sfdora.org/read/). The GARCIA working paper, developed under the EU’s 7th Research Framework Programme, listed a number of recommendations to combat the “leaky pipeline” phenomenon in academic careers, including drawing attention to the stabilization of post-doctoral positions, placing greater emphasis on work-life balance issues, and addressing the gendered context of academia [35]. The working paper also suggests that further research is needed on what hinders women’s career progression and on what makes it easier for men to progress in certain disciplines (STEM) than in others such as the social sciences and humanities.

**Box 1. Measures to reduce the gender gap in publication and, more generally, the inequalities faced by women in academic medicine**

**Table:** 

Promoting gender equality policies	Institutions and funding agencies should implement and enforce policies that explicitly promote gender equality in academic publishing. These policies can include measures such as targeted funding for female researchers, mentorship programs, and initiatives to increase the visibility of women’s research contributions.
Addressing implicit biases	Awareness programs and implicit bias training should be implemented within academic institutions to raise awareness about gender biases that may affect publication decisions. Reviewers and editors should be trained to evaluate research based on rigorous scientific inquiry free of implicit, unconscious bias. Double-blind peer review can mitigate the impact of gender bias.
Supporting work-life balance	Creating supportive environments that prioritize work-life balance can help alleviate the disproportionate burden on women in academia. Offering flexible working arrangements, parental leave policies, and childcare support can enable women to balance their professional and personal responsibilities, leading to increased productivity and opportunities for publication.
Mentorship and networking opportunities	Establishing mentorship programs that pair senior female researchers with early-career women can provide guidance, support, and networking opportunities. These programs can help overcome barriers to publication by offering advice on manuscript preparation, navigating the peer-review process, and fostering collaborations.
Recognition of diverse research contributions	Encouraging the recognition of diverse research contributions beyond traditional metrics, such as citation counts, can help address biases in academic publishing. This can be achieved by valuing different types of research outputs, including interdisciplinary work and contributions to open access publications (see DORA initiative presented in the main text).
Collaboration and knowledge sharing	Encouraging collaboration and knowledge sharing among researchers from different countries and regions can help address the disparities in academic publishing. International collaborations, joint research projects, and conferences can foster diverse perspectives, enhance research quality, and increase opportunities for publication.
Long-term monitoring and evaluation	Continuously monitoring and evaluating gender disparities in academic publishing is essential to measure progress and identify areas that require further attention. Longitudinal studies can track changes over time, providing valuable insights into the effectiveness of interventions and identifying persistent challenges.

In summary, gender policies and institutional initiatives are essential to address the low representation of women in academic careers, as measured here by their appearance as first or last author. However, as a socio-cultural concept, gender is constructed in everyday practices where women and men interact, including in academic settings. Research is needed to understand how gender is constructed and how it produces inequalities in context, intersecting with other dimensions such as class and race, to enable the formulation of context-specific measures.

### Limitations

Gender was inferred using a gender detection tool. However, NamSor was shown to lead to accurate results [28], and our results were similar using the subsamples (sensitivity analyses). In addition, gender determination based on first names raises ethical issues by oversimplifying a complex concept (gender). For example, it is not possible to assess non-binary or transgender identity using this method. In future studies evaluating gender inequalities in academic publication, researchers should consider adopting self-identification as the preferred method for obtaining the gender information of authors. Gender detection tools may introduce inherent biases, misclassifications, and oversimplifications by attempting to assign individuals into rigid male and female categories. Gender is a multifaceted and complex aspect of human identity, encompassing a wide spectrum of experiences and expressions. Self-identification respects individuals’ autonomy and self-perception, reducing the risk of misrepresenting or excluding those who do not fit within traditional gender frameworks. This approach fosters inclusivity, accuracy, and a deeper understanding of gender disparities in academic publishing.

## Conclusion

We found that the proportion of female authors in high-impact general internal medicine journals varied widely by country and region, and was lowest in Asia, particularly Japan, and the Middle East. The main limitation of the study is that gender was determined using a gender detection tool and not by self-identification, which may have introduced biases and failed to fully capture the complexity of gender identity. Further research should investigate the underlying factors and mechanisms contributing to gender disparities in academic publishing and assess the impact of these disparities on research outcomes and collaboration patterns. We recommend the adoption of self-identification as the preferred method for obtaining gender information in future studies. By fostering inclusivity, combating discrimination, and tracking progress over time, we can create an environment that maximizes the potential of all researchers, regardless of their gender.

## Supporting information

S1 Checklist STROBE statement—checklist of items that should be included in reports of *cross-sectional studies*. (DOCX)
